# A cohort study of maternal cardiometabolic risk factors and primary cesarean delivery in an integrated health system

**DOI:** 10.1371/journal.pone.0199932

**Published:** 2018-07-03

**Authors:** Monique M. Hedderson, Fei Xu, Sneha B. Sridhar, Emily S. Han, Charles P. Quesenberry, Yvonne Crites

**Affiliations:** 1 Division of Research, Kaiser Permanente Northern California, Oakland, California, United States of America; 2 The Kaiser Permanente Northern California Medical Group, Oakland, California, United States of America; Univesity of Iowa, UNITED STATES

## Abstract

**Background:**

Maternal cardiometabolic risk factors (i.e., hyperglycemia, pre-existing hypertension and high body mass index) impact fetal growth and risk of having a cesarean delivery. However, the independent and joint contribution of maternal cardiometabolic risk factors to primary cesarean section is unclear. We aimed to elucidate the degree to which maternal cardiometabolic risk factors contribute to primary cesarean deliveries and whether associations vary by infant size at birth in an integrated health system.

**Methods:**

A cohort study of 185,045 singleton livebirths from 2001 to 2010. Poisson regression with robust standard errors provided crude and adjusted relative risks (RR) and 95% confidence intervals (CIs) for cesarean delivery risk associated with risk factors. We then estimated the proportion of cesarean sections that could be prevented if the cardiometabolic risk factor in pregnant women were eliminated (the population-attributable risk [PAR]).

**Results:**

In a single multivariable model, maternal cardiometabolic risk factors were independently associated with cesarean delivery: RR (95% CI) abnormal glucose screening 1.04 (1.01–1.08); gestational diabetes 1.18 (1.11–1.18) and pre-existing diabetes 1.60 (1.49–1.71); pre-existing hypertension 1.16 (1.10–1.23); overweight 1.27 (1.24–1.30); obese class I 1.46 (1.42–1.51); obese class II 1.73 (1.67–1.80); and obese class III 1.97 (1.88–2.07); adjusting for established risk factors, medical facility and year. The associations between maternal cardiometabolic risk factors and primary cesarean delivery remained among infants with appropriate weights for gestational age. The PARs were 17.4% for overweight/obesity, 7.0% for maternal hyperglycemia, 2.0% for pre-existing hypertension and 20.5% for any cardiometabolic risk factor.

**Conclusions:**

Maternal cardiometabolic risk factors were independently associated with risk of primary cesarean delivery, even among women delivering infants born at an appropriate size for gestational age. Effective strategies to increase the proportion of women entering pregnancy at an optimal weight with normal blood pressure and glucose before pregnancy could potentially eliminate up to 20% of cesarean deliveries.

## Introduction

Primary cesarean delivery rates in the United States increased from 14.6% of births in 1996[1] to 21.9% in 2012[2]. Cesarean sections are now the most common surgery performed in the United States. This rapid increase occurred despite the fact that cesarean delivery increases the risk of adverse perinatal outcomes, including surgical complications, severe hemorrhage, puerperal infection and cardiac arrest[3] as well as complications requiring admission to the neonatal intensive care unit, obstetrical care related costs and the likelihood of delivery by cesarean section in subsequent births[4, 5]. A 2011 Joint Commission report determined that there are no data supporting that higher rates of cesarean sections improve perinatal outcomes, and yet the rates continue to rise[6]. Thus, reducing primary cesarean deliveries is essential to reduce the related morbidities. Studies are needed on potentially modifiable risk factors associated with primary cesarean delivery to inform future interventions to reduce rates of cesarean deliveries[6].

A growing body of literature is showing the multifactorial reasons for the rise in cesarean sections. Increases in obesity[7–9], pre-gestational diabetes[10], gestational diabetes mellitus (GDM)[11], and hypertension, for example, have occurred in parallel to the rise in primary cesarean section deliveries in recent decades, which provides evidence that adverse maternal cardiometabolic profile in pregnancy contributes to the high rates of primary cesarean section delivery. While several studies have examined the separate effects of obesity, glucose intolerance, and hyptetension in relation to cesarean section[12–17], the independent and joint effects of these cardiometabolic risk factors is unclear. In addition, while these maternal cardiometabolic risk factors impact fetal growth[18, 19], whether the impact of these risk factors on risk of cesarean delivery varies by infant size for gestational age remains unclear.

This cohort study examined the independent and joint associations between maternal cardiometabolic risk factors (BMI, pregnancy glycemia and pre-existing hypertension) and primary cesarean delivery overall and by infant size for gestational age in a racially-ethnically diverse cohort of 185,045 women delivering in Kaiser Permanente Northern California (KPNC) from 2001 to 2010. Our overall aim was to elucidate the degree to which maternal cardiometabolic risk factors contribute to primary cesarean deliveries, and whether these associations vary by infant size at birth in an integrated health care system with uniform access to care and finally to quantify the extent to which we could reduce the rates of cesarean section if we improved maternal cardiometabolic risk factors at a population level.

## Materials and methods

Kaiser Permanente Northern California (KPNC) provides comprehensive medical services through 14 delivery hospitals and 23 outpatient clinics to over 3 million members located in a 14-county region of Northern California. KPNC membership is well-representative of the population living in the geographical area served by this large, integrated health care delivery system, except that the KPNC population has slightly lower representation at the extremes of income[20]. As KPNC is an integrated health care delivery system, all pregnant women have access to care in a setting where the cost structure does not incentivize cesarean sections.

We identified 204, 231 singleton live births to women aged 15–45 between 2001 and 2010 among women without a prior cesarean section. We then matched this cohort to the California birth certificate record (99% successful linkage). We excluded women whose gestational age was less than 24 weeks or greater than 45 weeks (N = 3,274). We also excluded women for whom we were unable to verify whether the delivery resulted in a vaginal birth or cesarean delivery (N = 1,553). To confirm the delivery was a primary cesarean section, we first used ICD-9 code 654.2x to identify whether women had a prior cesarean, which we confirmed by verifying that the California state birth certificate was coded as a repeat cesarean. We excluded 2,059 deliveries for whom we were unable to confirm if they had a prior cesarean delivery consistently between these two sources. We also excluded deliveries at medical facilities that were not in existence during the entire study period (N = 12,300), leaving us with a final analytic cohort of 185,045. Of these, there were 152,011 unique women in the cohort due to the inclusion of repeat pregnancies.

Cesarean delivery was based on ICD-9 codes available in the EMR: 74.xx (excluding 74.3 and 74.91) and ICD-9 diagnosis: 669.7. Primary cesarean deliveries identified by ICD-9 codes were also verified against the California birth certificate record and the KPNC infant cohort database[21] (~98.9% were validated based on at least two sources).

We obtained maternal age at delivery, height, pregnancy body weight (assessed on average at 17 weeks’ gestation, with a range of 6–26 weeks’ gestation), and gestational age at the weight measurement from electronic medical records (EMR).

We searched the KPNC Diabetes Registry[22] to identify women with pre-existing diabetes. We then searched the KPNC Pregnancy Glucose Tolerance Registry[11], which classifies women without pre-existing diabetes by glucose values obtained from a 50-g glucose challenge test (hereafter referred to as the screening test). Women with an abnormal result (≥140 mg/dl) receive a follow up 100-g, 3-hour oral glucose tolerance test (hereafter referred to as the diagnostic test). At KPNC, 94% of women delivering live-born singletons undergo the recommended screening test for GDM[11].

We classified women as having GDM if two or more of the four plasma glucose values obtained during the 3-hour diagnostic test were abnormal according to the Carpenter-Coustan plasma glucose thresholds for GDM (fasting, 95 mg/dl; 1-hour, 180 mg/dl; 2-hour, 155 mg/dl; 3-hour, 140 mg/dl) or if there was an outpatient diagnosis of GDM[23]. Abnormal screening was defined as women having an abnormal screening test (≥140 mg/dl), but a normal diagnostic test (one or fewer abnormal values on the 3-hour diagnostic test).

We calculated pregnancy BMI as the maternal weight (kilograms) divided by height (meters) squared. BMI categories were created based on the World Health Organization International Classification: underweight (<18.5 kg/m^2^), normal (18.5–24.99 kg/m^2^), overweight (25.0–29.99 kg/m^2^), obese (30.00–34.99 kg/m^2^), obese class II (35.0 kg/m^2^-39.9 kg/m^2^), and obese class III (40.0 kg/m^2^ or higher)[24].

### Covariates

#### Infant size for gestational age

We defined large for gestational age (LGA) as birthweight greater than the 90^th^ percentile and small for gestational age (SGA) as birthweight less than the 10^th^ percentile, both according to the study population’s race-ethnicity and gestational age-specific birth weight distribution.

We obtained maternal education and race-ethnicity from the California birth certificate. We identified the following potential indications for cesarean section: obstructed labor using ICD-9 codes: 660.0,660.3 and 660.4; fetopelvic disproportion using ICD-9 codes: 653.4–653.9; breech position using ICD-9 codes 652.2 and Placenta previa using ICD-9 codes 641.0 and 641.1. Pre-existing hypertension as essential hypertension; hypertension secondary to a chronic underlying condition; pre-eclampsia or eclampsia superimposed on pre-existing hypertension; and unspecified hypertension of pregnancy (ICD-9 codes 642.0, 642.1,642.2 and 642.7).

### Statistical analysis

Univariable and multivariable Poisson regression with robust standard errors were used to provide crude and adjusted estimates of relative risks (RR) and 95% CIs for cesarean delivery risk associated with maternal cardiometabolic factors[25]. RR estimates were adjusted for maternal age at delivery, race-ethnicity, maternal education, parity, and medical facility to account for possible changes in the demographics and underlying risk factor for cesarean deliveries in the study population. We calculated partial population attributable risks (PAR) for being overweight or obese during pregnancy by the method described by Spiegelman and colleagues [26], using a publicly-available SAS Macro http://www.hsph.havard.edu/faculty/spiegelman/par.html). We used the KPNC population data to estimate the prevalence of GDM for the PAR calculations. We examined effect modification between maternal cardiometabolic risk factors and infant size for gestational age and all were significant (p-interaction BMI = 0.001, maternal glucose tolerance status: 0.003 and hypertension 0.010). Therefore, we conducted a stratified analysis by infant size for gestational age to see if the associations with maternal cardiometabolic risk factors were independent of infant size. We used SAS version 9.3 (SAS Institute Inc., Cary, NC) for all analyses.

The Kaiser Permanente Northern California Institutional Review Board and the State of California Committee for the Protection of Human Subjects approved this study and waived the requirement for obtaining written informed consent from study participants. Patient records were anonymized and de-identified prior to analysis.

## Results

The cohort was racially and ethnically diverse with more than 63% from non-white racial ethnic minority groups and over half of the women were overweight or obese, half of women were nulliparous and just over one third of women had a college education or higher (Table 1).

**Table 1:** Demographic characteristics by calendar year: Kaiser Permanente Northern California, 2001–2010^1^.

*Maternal/infant factor*	N = 185,045
	n (%)
**Primary cesarean rate**	30,347 (16.4)
**Age at delivery (years)**	
Mean ± SD	29.0 ± 5.5
**Race/Ethnicity**	
Non-Hispanic White	67,922 (36.8)
Hispanic	51,533 (27.9)
Asian	41,856 (22.7)
Black	14,114 (7.7)
Other	9,079 (4.9)
**Pregnancy body mass index (kg/m**^**2**^**)**	
< 18.5 (Underweight)	2,396 (1.3)
18.5–24.9 (Normal weight)	81,117 (43.8)
25.0–29.9 (Overweight)	57,899 (31.3)
30.0–34.9 (Obese class I)	26,160 (14.1)
35.0–39.9 (Obese class II)	11,131 (6.0)
≥ 40 (Obese class III)	6,342 (3.4)
**Glucose tolerance**	
Normal screening	140,781 (78.5)
Abnormal screening and Normal 3-h OGTT	20,780 (11.6)
Gestational diabetes	16,546 (9.2)
Pre-existing diabetes	1,304 (0.7)
**Hypertension status**	
None	162,673 (89.9)
Pre-existing hypertension	4,190 (2.3)
Gestational hypertension	6,226 (3.4)
Preeclampsia/Eclampsia	7,860 (4.3)
**Infant birth weight (grams)**	
Mean ± SD	3,395.7 ± 545.9
**Size for gestational age**	
Small for gestational age	18,154 (9.8)
Appropriate for gestational age	150,311 (81.2)
Large for gestational age	16,575 (9.0)
**Fetopelvic disproportion**	3,590 (2.0)
**Obstructed Labor**	9,159 (5.1)
**Placenta Previa**	1,526 (0.8)
**Breech Position**	10,950 (6.1)

^1^ Not reported, missing, and unknown are as follows: Race: n = 541, Glucose tolerance: n = 5,634, size for gestational age: n = 5 and n = 4096 women were missing all hospital discharge diagnoses codes and were missing information on hypertension, fetopelvic disproportion, obstructed labor, placenta previa, and breech position.

Several maternal cardiometabolic risk factors appear to independently contribute to risk of cesarean delivery above and beyond other established risk factors. Table 2 shows the relative risks (RRs) and 95% CIs from a single multivariable model containing all cardiometabolic risk factors; the risk of having a cesarean delivery increased along with maternal BMI, increasing glucose intolerance, and maternal hypertension. All maternal cardiometabolic risk factors were significantly and independently associated with having a cesarean section delivery. These associations remained significant after further adjusting for other indications for cesarean delivery: fetopelvic disproportion, obstructed labor, placenta previa and breech position (Table 2). In addition, women delivering either SGA [RR 1.12 (95% CI:1.09–1.16)] or LGA [RR 1.44 (95% CI: 1.40–1.49)] infants also had an increased risk of cesarean delivery when compared with women delivering appropriate size for gestational age infants (Table 2). We found a trend of increasing risk of cesarean delivery with increasing number of cardiometabolic risk factor (RR:0 risk factors = reference group, 1 risk factor 1.37 (95% CI, 1.34–1.41), 2 risk factors 1.69 (95% CI, 1.64–1.74), and 3+ risk factors (2.34 (95% CI, 2.19–2.51); *P-* trend = <0.01).

**Table 2:** Risk Ratios (RRs) and 95% confidence intervals (CIs) for primary cesarean delivery associated with maternal and infant factors.

*Maternal/infant factor*	RR (95% CI)	
**Year of delivery**	Model 1^1^	Model 2^2^
2001	1.00 (ref)	1.00 (ref)
2002	1.04 (0.98–1.10)	1.05 (0.99–1.11)
2003	1.02 (0.96–1.08)	1.05 (0.99–1.11)
2004	1.04 (0.99–1.10)	1.10 (1.04–1.16)
2005	1.08 (1.02–1.14)	1.15 (1.09–1.21)
2006	1.08 (1.03–1.14)	1.21 (1.15–1.28)
2007	1.10 (1.04–1.16)	1.29 (1.23–1.36)
2008	1.11 (1.06–1.17)	1.29 (1.23–1.36)
2009	1.08 (1.02–1.13)	1.23 (1.17–1.30)
2010	1.00 (0.95–1.06)	1.16 (1.10–1.22)
**Pregnancy body mass index (kg/m^2^)**		
< 18.5 (underweight)	0.79 (0.70–0.89)	0.79 (0.71–0.88)
18.5–24.9 (normal weight)	1.00 (ref)	1.00 (ref)
25.0–29.9 (overweight)	1.26 (1.23–1.30)	1.26 (1.23–1.29)
30.0–34.9 (obese class I)	1.46 (1.42–1.51)	1.43 (1.38–1.47)
35.0–39.9 (obese class II)	1.72 (1.65–1.79)	1.68 (1.62–1.75)
≥ 40 (obese class III)	1.98 (1.89–2.07)	1.90 (1.81–1.99)
**Glucose tolerance**		
Normal screening	1.00 (ref)	1.00 (ref)
Abnormal screening and normal 3-hour OGTT	1.05 (1.02–1.08)	1.04 (1.01–1.07)
Gestational diabetes	1.16 (1.13–1.20)	1.15 (1.11–1.18)
Pre-existing diabetes	1.53 (1.43–1.63)	1.47 (1.37–1.59)
**Hypertension status**		
No hypertension	1.00 (ref)	1.00 (ref)
Gestational hypertension	1.18 (1.12–1.23)	1.15 (1.10–1.21)
Pre-existing hypertension	1.28 (1.22–1.35)	1.23 (1.16–1.30)
Preeclampsia / Eclampsia	1.58 (1.53–1.64)	1.53 (1.47–1.58)
**Size for gestational age**		
Small for gestational age	1.07 (1.04–1.11)	1.09 (1.06–1.13)
Appropriate for gestational age	1.00 (ref)	1.00 (ref)
Large for gestational age	1.68 (1.64–1.73)	1.44 (1.40–1.49)

^1^ Adjusted for maternal age at delivery, race/ethnicity, education level, medical facility of delivery, and parity.

^2^ Adjusted for maternal age at delivery, race/ethnicity, education level, medical facility of delivery, parity, obstructed labor status, fetopelvic disproportion status, placenta previa status, and breech position status.

**Fig 1:**
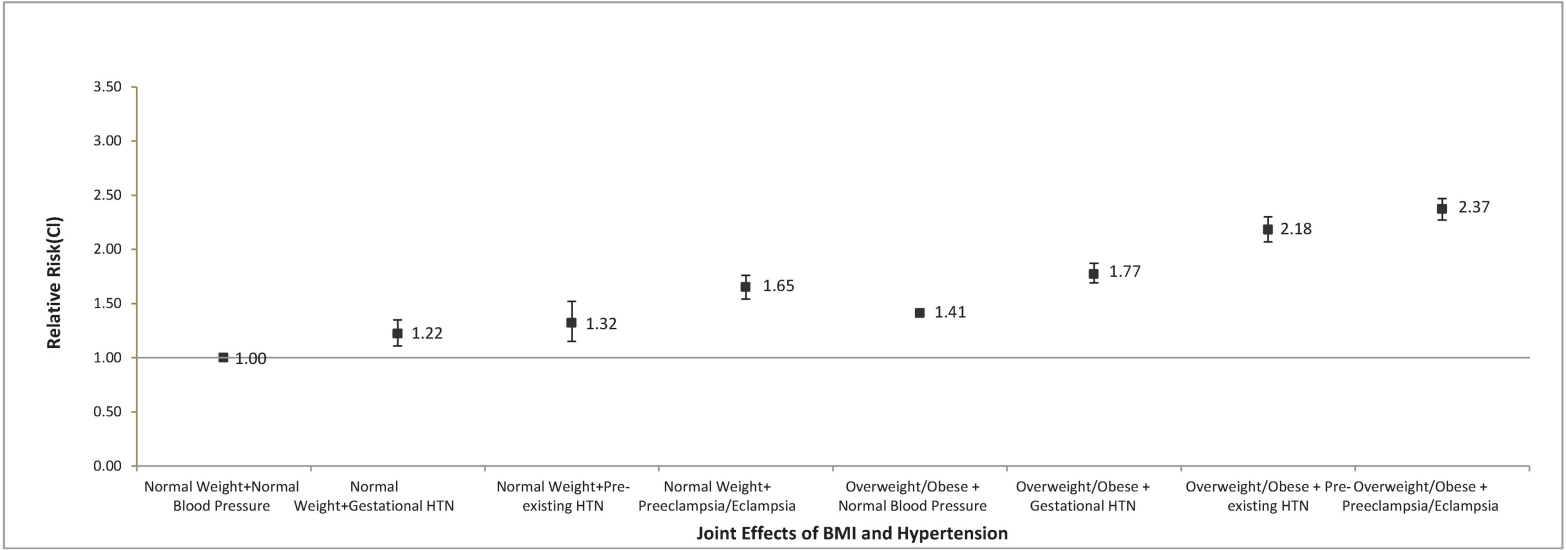
The joint effects of maternal BMI on maternal pre-existing hypertension in relation to primary cesarean delivery.

**Fig 2:**
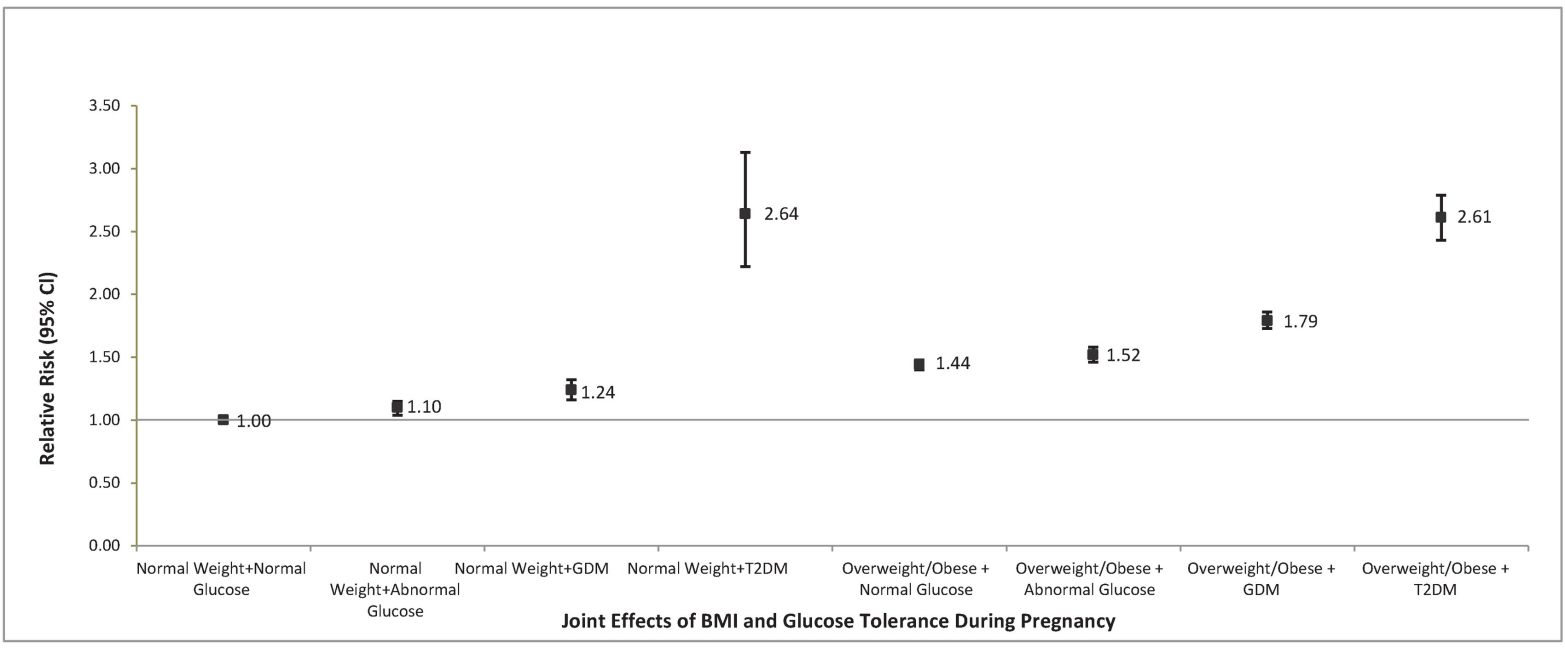
The joint effects of maternal BMI and maternal glucose tolerance status and primary cesarean delivery.

Maternal overweight/obesity synergistically increases the risk of cesarean delivery above and beyond maternal glucose intolerance and pre-existing hypertension. The joint effects of maternal hypertension and maternal overweight/obesity are reported in Fig 1. Compared to low-risk women with a BMI <25.0 kg/m^2^ and normal blood pressure, women with a BMI < 25.0 with pre-existing hypertension had a 28% increased risk, overweight/obese women with normal blood pressure had a 45% increased risk, and overweight/obese women with pre-existing hypertension had a 2-fold increased risk of having a primary cesarean section. The independent and joint effects of maternal glucose tolerance status and maternal overweight/obesity are reported in Fig 2. For all degrees of glucose intolerance status in pregnancy, the risk of requiring a cesarean delivery was higher among overweight and obese pregnant women. For women with pre-existing diabetes, we found a 2.5 fold increased risk of requiring cesarean section that was independent of maternal BMI (Fig 2).

The increased risk for cesarean delivery appears to occur even among infants who are appropriate for gestational age (Table 3). The relative risks for having a cesarean section associated with pre-pregnancy BMI among women whose infants were appropriate size for gestational age were higher than for women whose infants were large for gestational age. These rates were not different than the risk ratios for women whose infants were small for gestational age, suggesting cesarean sections associated with women with high pregnancy BMIs are occurring even among women whose infants are appropriate size. Similarly, even among women whose infants were appropriate for gestational age, the risk ratio associated with gestational diabetes was 1.16 (95% CI: 1.12–1.20).

**Table 3:** Risk Ratios (RRs)^1^ and 95% confidence intervals (CIs) for primary cesarean delivery associated with maternal cardiometabolic risk factors, stratified by infant size at delivery.

	SGA	AGA	LGA
Maternal Cardiometabolic Risk Factor			
**Pregnancy body mass index (kg/m^2^)**			
< 18.5 (underweight)	0.79 (0.61–1.02)	0.80 (0.70–0.92)	0.84 (0.48–1.47)
18.5–24.9 (normal weight)	1.00	1.00	1.00
25.0–29.9 (overweight)	1.29 (1.20–1.39)	1.27 (1.23–1.30)	1.19 (1.11–1.28)
30.0–34.9 (obese class I)	1.51 (1.37–1.66)	1.47 (1.42–1.53)	1.34 (1.24–1.45)
35.0–39.9 (obese class II)	1.72 (1.52–1.95)	1.75 (1.67–1.84)	1.49 (1.36–1.64)
≥ 40 (obese class III)	2.13 (1.85–2.44)	2.07 (1.96–2.18)	1.61 (1.46–1.78)
**Glucose tolerance**			
Normal screening	1.00	1.00	1.00
Abnormal screening/normal 3-hour OGTT	1.08 (0.98–1.19)	1.04 (1.00–1.08)	1.09 (1.01–1.17)
Gestational diabetes	1.07 (0.96–1.18)	1.14 (1.10–1.19)	1.33 (1.25–1.42)
Pre-existing diabetes	1.42 (1.10–1.83)	1.53 (1.40–1.67)	1.70 (1.52–1.89)
**Hypertension status**			
No hypertension	1.00		
Gestational hypertension	1.23 (1.08–1.39)	1.19 (1.12–1.25)	1.09 (0.99–1.21)
Preeclampsia / Eclampsia	1.81 (1.67–1.96)	1.58 (1.51–1.65)	1.29 (1.18–1.41)
Pre-existing hypertension	1.52 (1.34–1.72)	1.28 (1.20–1.35)	1.08 (0.95–1.23)

^1^ Adjusted for year of delivery, maternal age at delivery, race/ethnicity, education level, medical facility of delivery and parity.

The population attributable risks suggest that the percentages of cesarean deliveries that could be prevented if maternal cardiometaobolic risk factors were eliminated were 17.4% for overweight/obesity, 7.0% for hyperglycemia, 2% for hypertension and 20.5% for any cardiometabolic risk factor.

## Discussion

We found adverse maternal cardiometabolic risk factors including increasing degree of pregnancy glucose intolerance status, pre-existing hypertension and maternal overweight or obesity were all independent risk factors for primary cesarean delivery, after adjusting for other established risk factors for cesarean delivery. Maternal glucose intolerance status and pre-existing hypertension both synergistically increased the risk of primary cesarean section beyond the risk associated with maternal overweight/obesity alone. The estimated population attributable risk for having any cardiometabolic risk factor was 20.5% and the population attributable risk for maternal overweight/obesity it was 17%. The high population-attributable risk for high BMI is driven by the high prevalence of overweight/obesity among pregnant women and highlights the need for strategies to decrease overweight and obesity among reproductive-aged women.

The risk of requiring a cesarean delivery increased with increasing BMI category and was highest among obese class III women. These findings are consistent with prior studies which suggested there is an increased risk of cesarean delivery with increasing BMI category[12–15]. Surprisingly, we found the relative risk of cesarean section among obese class III women was highest among women whose infants were either small or appropriate for gestational age, suggesting this effect is independent of excess fetal growth. The reason underlying the increased risk of cesarean delivery among severely obese women occurring even when the infant is normal or small size may include the practice of “defensive medicine”[27] for fear of malpractice lawsuits, biologic risk factors, or other maternal medical indications for a planned cesarean section. For example, it has been suggested that obese women may have more labor complications due to having a longer active labor phase and decreased cervical dilation rate which may prompt providers to perform a cesarean delivery[28, 29].

We found that maternal pre-existing hypertension was significantly associated with an increased risk of cesarean delivery independent of other known risk factors and indications. However, the population attributable risk associated with pre-existing hypertension was only 2.0% compared with 17.0% for overweight/obesity, suggesting that while pre-existing hypertension is risk factor for requiring a primary cesarean delivery, at the population level they are not accounting for a large a percentage of cesarean deliveries compared to maternal overweight and obesity since the overall prevalence of pre-existing hypertension among pregnant women is still low. Recently, a large study of 123 million singleton deliveries in the United States (1979–2010) found period effects in primary cesarean increasing after 1997 that were explained by a combination of trends in obesity and chronic hypertension, as well as demographic shifts over time[30].

A prior study of 42,071 singleton births in South Carolina found that both pre-existing diabetes and GDM increased the risk of cesarean delivery independently of their effects on birth weight[16]; however, they lacked information on maternal BMI and objectively measured glucose levels during pregnancy. Another study of women undergoing a labor trial found that both prepregnancy obesity and diabetes independently increased the risk for cesarean delivery[17]. We were able to look at increasing degree of objectively measured maternal glucose tolerance status and found a trend of increasing risk of primary cesarean delivery with increasing degree of maternal glucose intolerance. We observed a modest increased risk of primary cesarean section among women with GDM even when infants were appropriate for gestational age. This is similar to a finding in the landmark study by Naylor et al which found that clinicians performed cesarean sections more often among women diagnosed with GDM, even in the absence of excess fetal growth[31]. Further research is required to understand the increased primary cesarean section rates among women with GDM and mild hyperglycemia even when delivering appropriate size for gestational age infants. We also found a significant increased risk of primary cesarean section among women whose infants were born appropriate size for gestational age associated with maternal overweight and obesity and hypertension. This suggests that the associations between maternal cardiometabolic risk factors and primary cesarean section are not due exclusively to the excess or restricted fetal growth associated with the risk factors.

The strengths of this study include the large racially, ethnically, and socioeconomically diverse population of women, the integrated health care delivery system which ensures access to medical care, and the electronic health record which provides high quality data. This enabled us to look at the contribution of maternal cardiometabolic risk factors to risk of primary cesarean deliveries. In addition, in KPNC there is no cost incentive to perform additional cesarean sections. However, the study also had limitations. We were unable to determine whether the cesarean section deliveries were planned or emergency. KPNC may not be generalizable to the extremes of the income distribution which are slightly under-represented in the KPNC membership [32, 33]; the findings may also not be generalizable to other health systems with different practice guidelines or to the uninsured population. We lacked more detailed measures of quality of obstetric care, such as provider characteristics which might have helped us better understand the observed associations. Future studies are needed to determine if the increased performance of cesarean deliveries among women with cardiometabolic risk factors improves health outcomes for the mother and the baby or if the recognition of these conditions causes clinicians to have a lower threshold for performing cesarean deliveries without added benefit for mom and baby.

Maternal adverse cardiometabolic risk factors including overweight/obesity, glucose tolerance status and pre-existing hypertension were all independently associated with risk of cesarean delivery even after controlling for infant size for gestational age and other indications for having a cesarean delivery. Strategies to increase the proportion of women entering pregnancy at an optimal weight and to improve glucose and hypertension before and/or during pregnancy could potentially eliminate up to 20% of cesarean deliveries. Given that maternal cardiometabolic risk factors are associated with cesarean deliveries even when infants are appropriate for gestational age, it is important to further clarify the patient, clinician and health system-level factors contributing to primary cesarean deliveries, especially among women with adverse cardiometabolic risk profiles.
